# Supplementary material for: Formation of S. pombe Erh1 homodimer mediates gametogenic gene silencing and meiosis progression
Source: Sci Rep. 2020 Jan 23;10:1034. doi: 10.1038/s41598-020-57872-4 (PMC6978305; doi:10.1038/s41598-020-57872-4)
Supplement: Supplementary file 1 — Supplemental information. [file 41598_2020_57872_MOESM1_ESM.pdf]

Supplementary files

Formation of *S. pombe* Erh1 homodimer mediates gametogenic gene silencing and meiosis progression

Ditipriya Hazra[1,#], Vedrana Andrić[2,4,#], Benoit Palancade[3], Mathieu Rougemaille[2] and Marc Graille[1]

[1]: BIOC, CNRS, Ecole Polytechnique, Institut Polytechnique de Paris, F-91128 Palaiseau, France.
[2]: Institut de Biologie Intégrative de la Cellule, CNRS, UMR9198, Université Paris-Saclay, 91190 Gif-sur-Yvette, France
[3]: Institut Jacques Monod, CNRS, UMR7592, Univ Paris-Diderot, Sorbonne Paris Cité, 75013 Paris, France
[4]: Université Paris-Saclay, 91190 Gif-sur-Yvette, France
[#]: equal contribution
Correspondence should be addressed to MR (mathieu.rougemaille@i2bc.paris-saclay.fr) and MG (marc.graille@polytechnique.edu).

Materials and methods.

Size Exclusion Chromatography - Multi-Angle Laser Light Scattering (SEC-MALLS)

These analyses were performed as previously described in (van Tran, Ernst et al. 2019) using lysis buffer (20 mM Tris-HCl pH 7.5, 200 mM NaCl, 5 mM 2-mercaptoethanol).

CD

CD spectra were recorded using a Jobin-Yvon Mark VI circular dichrograph (scan speed of 0.2 nm/sec, integration time of 0.2 sec, delay spectra of 0 sec, slit width of 0.2 nm and bandwidth of 2 nm) using a quartz cuvette with a 0.1-cm path length at room temperature. Blanks (20mM Na/K phosphate buffer) were run before each spectrum and subtracted from the raw data. Five spectra were averaged. The final protein concentrations were 10 µM in 20mM Na/K phosphate buffer (pH 7.5).

Table S1: Oligonucleotides and plasmids used to over-express proteins in *E. coli*

ORF	Primer name	Primer sequence (the restriction sites are underlined)	Plasmid
Erh1	oMG511	TATAGGATCCAGCCCCCCACCCGCCG	pMG921
	oMG512	GCGGCTCGAGTTACGGAATCTGACGAGCCGC	
Erh1$_{I11R,L13R}$	oMG629	CGAATCTCATATCAGGCTGAGGATTCAGCAAGGTTCTGACCCT	pMG945
	oMG630	CCTTGCTGAATCCTCAGCCTGATATGAGATTCGGCGGGTGGGGGGC	
Mmi1-[95-122]	oMG605	TATA GGATCC GGTAAATATGATTTTAGCAGGC	pMG915
	oMG606	TATA CTCGAG TCAAGACTCACGACGAAGG	

Table S2: *S. pombe* strains used in this study

Strain	Genotype
PR040	h90, *ura4-DS/E, ade6-M210, leu1-32, mat3M::ura4+*
PR162	h90, *ura4-D18, ade6-M210, leu1-32, mat3M::ade6+*
PR808	PR040, *pREP41::LEU2*
PR1026	PR040, mei4*::nat^{R}MX* mmi1*::hph^{R}MX kan^{R}MX::*P_{nmt41}-TAP-Mei2 pREP41*::LEU2*
PR1316	PR040, mei4*::nat^{R}MX* mmi1*::hph^{R}MX* pREP41*::LEU2*
PR1413	PR040, *kan^{R}MX::*P_{nmt41}-TAP-Mei2 pREP41*::LEU2*
PR1414	PR040, erh1*::nat^{R}MX kan^{R}MX::*P_{nmt41}-TAP-Mei2 pREP41*::LEU2*
PR1415	PR040, erh1*::nat^{R}MX kan^{R}MX::*P_{nmt41}-TAP-Mei2 pREP41-GFP-Erh1*::LEU2*
PR1416	PR040, erh1*::nat^{R}MX kan^{R}MX::*P_{nmt41}-TAP-Mei2 pREP41-GFP-$Erh1_{I11R,L13R}$*::LEU2*
PR1420	PR040, erh1*::nat^{R}MX* pREP41*::LEU2*
PR1421	PR040, erh1*::nat^{R}MX* pREP41-GFP-Erh1*::LEU2*
PR1422	PR040, erh1*::nat^{R}MX* pREP41-GFP-$Erh1_{I11R,L13R}$*::LEU2*
PR1440	PR040, erh1*::nat^{R}MX* Mmi1-TAP*::hph^{R}MX* pREP41-GFP-Erh1*::LEU2*
PR1441	PR040, erh1*::nat^{R}MX* Mmi1-TAP*::hph^{R}MX* pREP41-GFP- $Erh1_{I11R,L13R}$*::LEU2*
PR1488	PR162, erh1*::kan^{R}MX* pREP41*::LEU2* pREP42X-2xFLAG-Erh1*::URA4*
PR1489	PR162, erh1*::kan^{R}MX* pREP41*::LEU2* pREP42X-2xFLAG-$Erh1_{I11R,L13R}$*::URA4*
PR1490	PR162, erh1*::kan^{R}MX* pREP41-TAP-$Erh1_{I11R,L13R}$*::LEU2* pREP42X-2xFLAG-$Erh1_{I11R,L13R}$*::URA4*
PR1502	PR040, Erh1-GFP*::kan^{R}MX*
PR1503	PR162, erh1*::kan^{R}MX* pREP41-TAP-Erh1*::LEU2* pREP42X-2xFLAG-Erh1*::URA4*

Table S3: Oligonucleotides used in this study

Primers	Sequence	Related figures
P249: *mei4*+ fwd	5’-TGGATCAGATCCGTGGAATC-3’	3C, 3D
P250: *mei4*+ rev	5’-AACGCTCGATTAGAAGGCAT-3’	3C, 3D
P253: *act1*+ fwd	5’-AACCCTCAGCTTTGGGTCTT-3’	3C, 3D
P254: *act1*+ rev	5’-TTTGCATACGATCGGCAATA-3’	3C, 3D
P325: *ssm4*+ fwd	5’-ACACAGTTTACGGGATTCTA-3’	3C, 3D
P326: *ssm4*+ rev	5’-GATTGTGATGAAAACTGGGT-3’	3C, 3D
P573: meiRNA fwd	5’-GGATGAATAGTAGCTTAGAT-3’	3C, 3D
P574: meiRNA rev	5’-GCTTTCAAGGATAACAATGC-3’	3C, 3D

Table S4: meiRNA probes for SmFISH

Probes	Sequence
#1	5’-ATACCCACTAAGTCTGTTTA-3’
#2	5’-CGGCAGAAGATTGACCAACA-3’
#3	5’-GCATATTCCGTCTTACAATA-3’
#4	5’-ACCAACTAAAGCGATCTTGC-3’
#5	5’-GACCATTTCAAAATGTTGCA-3’
#6	5’-TACCGAATCCAGCTTTTTGA-3’
#7	5’-CAGAGCTTAGAAGACAAGGT-3’
#8	5’-TAACTGGACCCCATCAAGAA-3’
#9	5’-TAAACCAACTTGGGGGTTGG-3’
#10	5’-TCTAAGCTACTATTCATCCA-3’
#11	5’-AGTAGATTCCATCAGTCATA-3’
#12	5’-TGCAGCCAAAAAGTGTACCA-3’
#13	5’-CATTGTAAGTGCTTTCAAGG-3’
#14	5’-TTCAGTCATTCGCAAAGTTT-3’
#15	5’-AGTCGTTTTATTTCTTTTCT-3’
#16	5’-GTTTCAACAATAGTTCAGGT-3’
#17	5’-TCTGTTTCAGGAATACGTTT-3’
#18	5’-TGTTTCGCATCAAACTTTCA-3’
#19	5’-GCGTTTAAACAAACTGCGGG-3’
#20	5’-TGGTTTCAGCACGTTTTCAA-3’
#21	5’-TTGGTTTGCAGGGTTTAACG-3’
#22	5’-CTTGCTGTGGTTATTGTTTA-3’

Supplementary Figure 1: Structural rearrangements of Erh1 upon binding to Mmi1

A. Superimposition of the three copies of Erh1 present in the asymmetric unit. All the panels from Fig. S1 have been generated using the Pymol software version 1.7.2.2 Schrödinger, LLC (http://www.pymol.org/).

B. Comparison of apo and Mmi1-bound Erh1 structures (rmsd values raging from 0.8-1.2Å over 80 Cα atoms). This reveals a large conformational change of the α2-β3 hinge characterized by a 4Å translation of Ile66 Cα atom and a 180° rotation of Tyr67 side chain (indicated by a red arrow). As a result, this renders accessible a hydrophobic cavity at the surface of Erh1, in which Phe99 side chain from one Mmi1 accommodates in the Erh1-bound structure. Concomitantly, Erh1 Tyr67 hydroxyl group forms an hydrogen bond (depicted as a black dashed line) with the Gly107 carbonyl group from the second Mmi1 peptide.

C. Upon Mmi1 binding, the N-terminal extremity of Erh1 strand β1 rearranges and His9 side chain flips by 180° (red arrow) to stack with Mmi1 Trp112 side chain.

Supplementary Figure 2: The Erh1 WT and I11R/L13R mutant proteins have an overall similar fold in solution according to circular dichroism.

Supplementary Figure 3: Erh1 does not contribute to Mmi1-dependent downregulation of Mei2

Western blot showing total TAP-Mei2 levels expressed from the P_{nmt41} promoter in strains of the indicated genotypes. anti-GFP and anti-CDC2 antibodies were used to evaluate Erh1 levels and loading, respectively.

Supplementary Figure 4: Uncropped gel images

Shown are full-size blots for Figure 2B, 2C, 2D, 2E and S3. Images displayed in the figures are surrounded in red.

References

van Tran, N., F. G. M. Ernst, B. R. Hawley, C. Zorbas, N. Ulryck, P. Hackert, K. E. Bohnsack, M. T. Bohnsack, S. R. Jaffrey, M. Graille and D. L. J. Lafontaine (2019). "The human 18S rRNA m6A methyltransferase METTL5 is stabilized by TRMT112." Nucleic Acids Res **47**(15): 7719-7733.

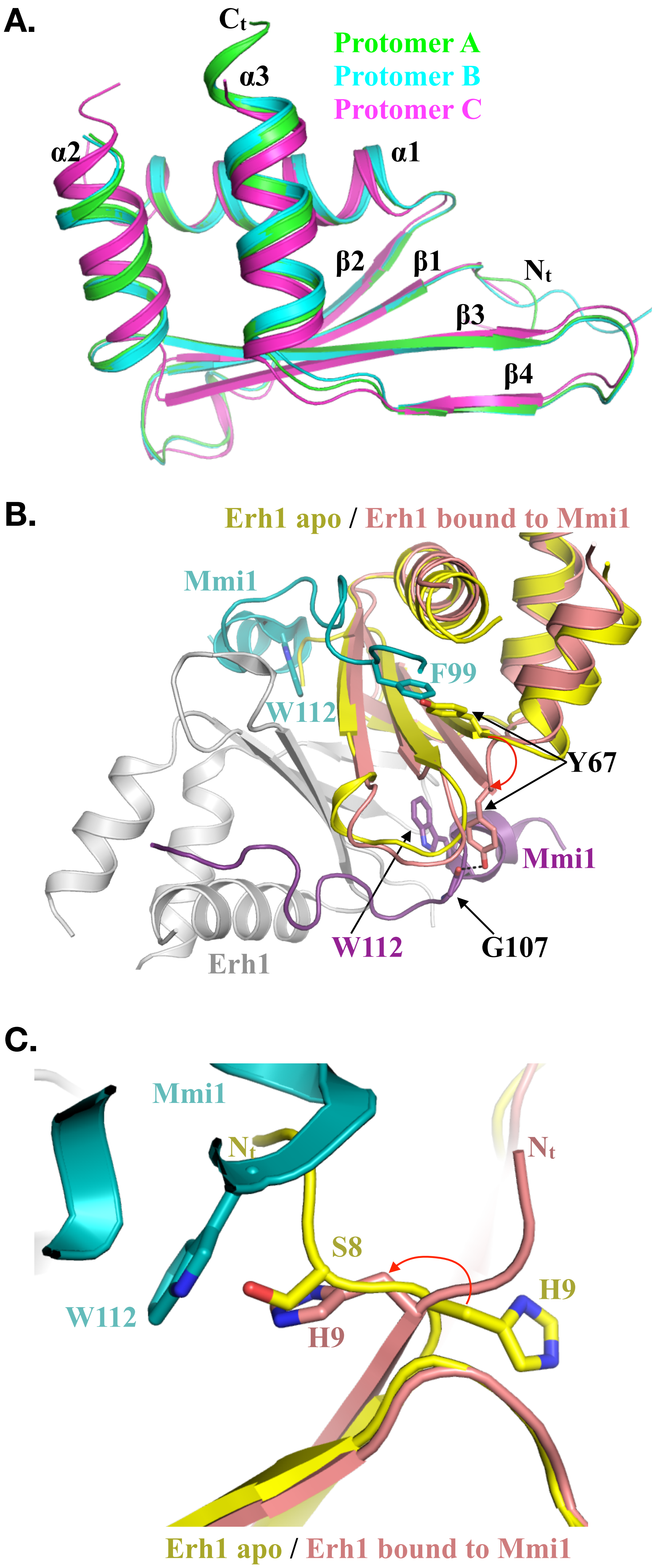

Figure S1: Structural rearrangements of Erh1 upon binding to Mmi1

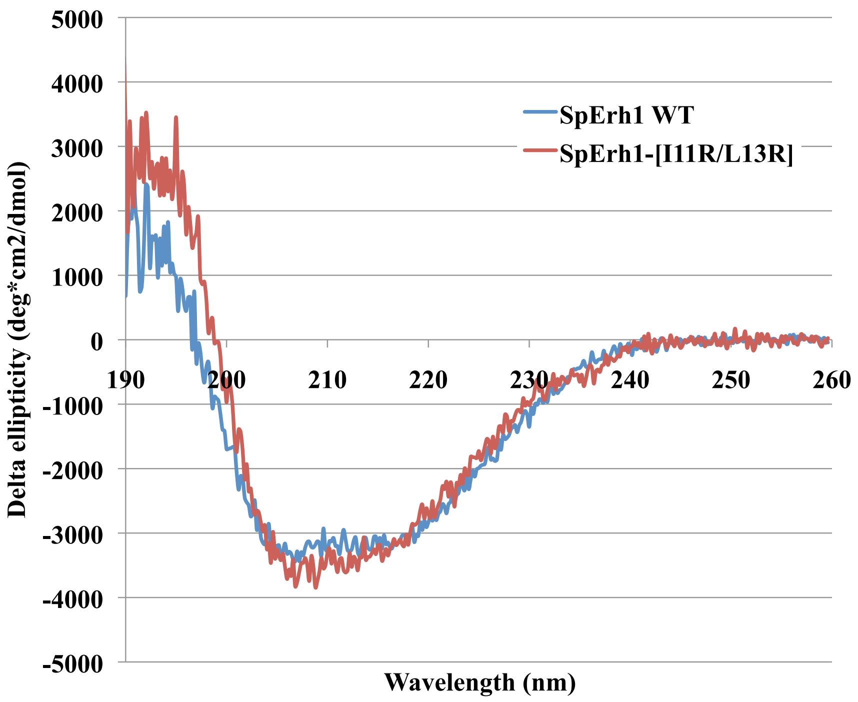

Figure S2: The *Sp*Erh1 double mutant is well-folded

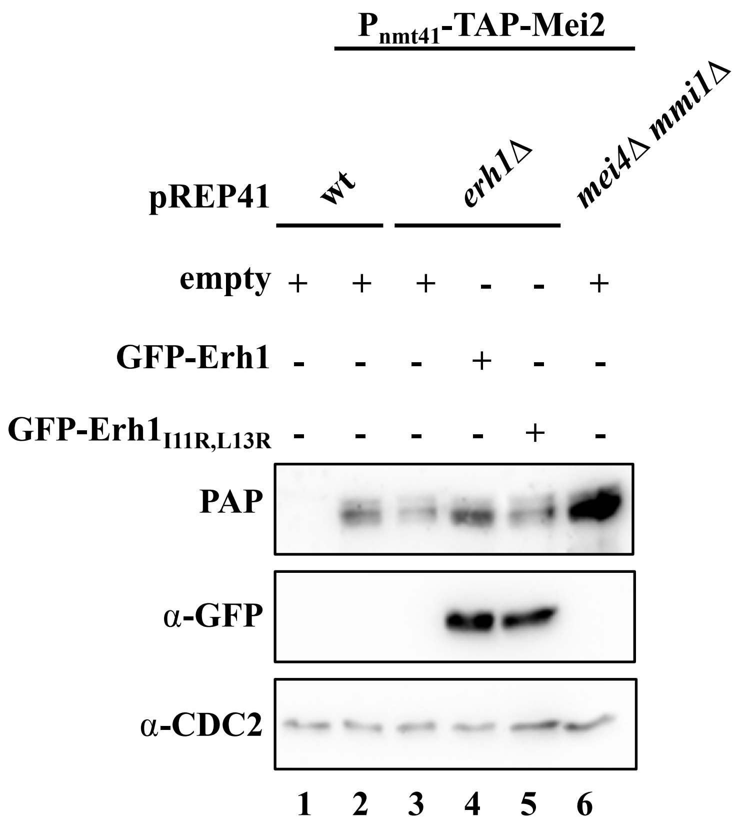

Figure S3: Erh1 does not contribute to Mmi1-dependent down-regulation of Mei2

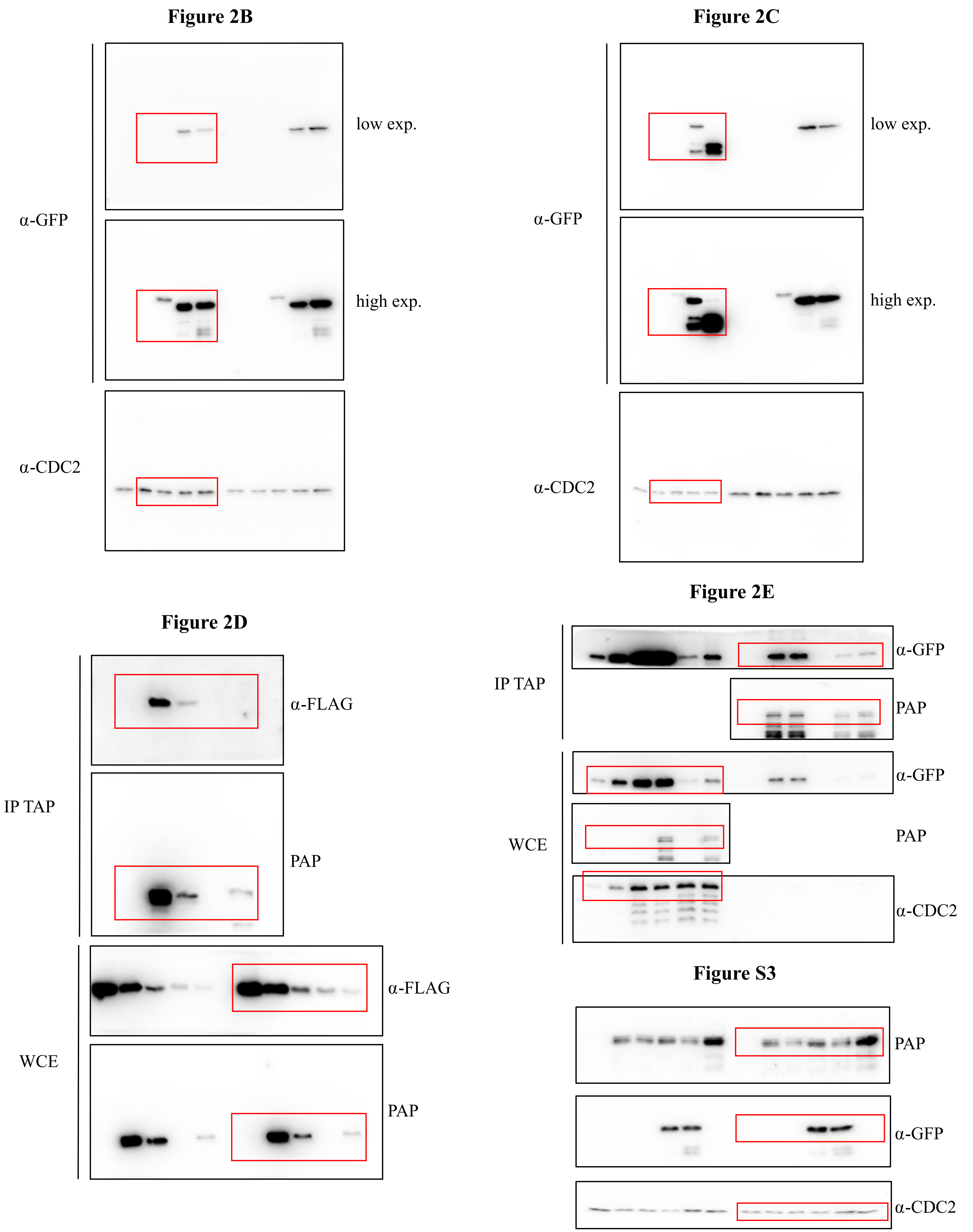

Figure S4: Uncropped gel images
